# Predictors and changes of physical activity in idiopathic pulmonary fibrosis

**DOI:** 10.1186/s12890-022-02134-4

**Published:** 2022-09-09

**Authors:** Diana Badenes-Bonet, Anna Rodó-Pin, Diego Castillo-Villegas, Vanesa Vicens-Zygmunt, Guadalupe Bermudo, Fernanda Hernández-González, Karina Portillo, Juana Martínez-Llorens, Roberto Chalela, Oswaldo Caguana, Jacobo Sellarés, Maria Molina-Molina, Xavier Duran, Joaquim Gea, Diego Agustín Rodríguez-Chiaradia, Eva Balcells

**Affiliations:** 1 Interstitial Lung Disease Unit, Respiratory Medicine Department, Hospital del Mar, Passeig Marítim 25, 08003 Barcelona, Spain; 2 Department of Medicine and Life Sciences, Universitat Pompeu Fabra (UPF), Barcelona, Spain; 3 Centro de Investigación en Red de Enfermedades Respiratorias, (CIBERES), Instituto de Salud Carlos III (ISCIII), Barcelona, Spain; 4 IMIM (Hospital del Mar Medical Research Institute), Barcelona, Spain; 5 Respiratory Medicine Department, Hospital de la Santa Creu i Sant Pau, Barcelona, Spain; 6 Respiratory Medicine Department, IDIBELL, Hospital Universitari de Bellvitge, Barcelona, Spain; 7 Respiratory Medicine Department, Hospital Clínic, Barcelona, Spain; 8 Respiratory Medicine Department, Hospital Germans Trias i Pujol, Barcelona, Spain; 9 Scientific, Statistics and Technical Department, Hospital del Mar-IMIM, Barcelona, Spain

**Keywords:** Physical activity, Idiopathic pulmonary fibrosis, Predictors, Muscle strength, Depression, Prognosis

## Abstract

**Background:**

Different clinical predictors of physical activity (PA) have been described in idiopathic pulmonary fibrosis (IPF), but studies are lacking evaluating the potential role of muscle strength and anxiety and depression symptoms in PA limitation. Moreover, little is known about the impact of changes in PA in the course of the disease. The aim of the present study was to investigate the relationship between baseline PA and a wide range of variables in IPF, to assess its longitudinal changes at 12 months and its impact on progression free-survival.

**Methods:**

PA was assessed by accelerometer and physiological, clinical, psychological factors and health-related quality of life were evaluated in subjects with IPF at baseline and at 12 month follow-up. Predictors of PA were determined at baseline, evolution of PA parameters was described and the prognostic role of PA evolution was also established.

**Results:**

Forty participants with IPF were included and 22 completed the follow-up. At baseline, subjects performed 5765 (3442) daily steps and spent 64 (44) minutes/day in moderate to vigorous PA. Multivariate regression models showed that at baseline, a lower six-minute walked distance, lower quadriceps strength (QMVC), and a higher depression score in the Hospital Anxiety and Depression scale were associated to lower daily step number. In addition, being in (Gender-Age-Physiology) GAP III stage, having a BMI ≥ 25 kg/m^2^ and lower QMVC or maximum inspiratory pressure were factors associated with sedentary behaviour. Adjusted for age, gender and forced vital capacity (FVC) (%pred.) a lower progression-free survival was evidenced in those subjects that decreased PA compared to those that maintained, or even increased it, at 12 months [HR 12.1 (95% CI, 1.9–78.8); *p* = 0.009].

**Conclusion:**

Among a wide range of variables, muscle strength and depression symptoms have a predominant role in PA in IPF patients. Daily PA behaviour and its evolution should be considered in IPF clinical assessment and as a potential complementary indicator of disease prognosis.

**Supplementary Information:**

The online version contains supplementary material available at 10.1186/s12890-022-02134-4.

## Introduction

Idiopathic pulmonary fibrosis (IPF) is a progressive fibrosing interstitial lung disease (ILD) with a high morbidity and mortality [1]. Exertional dyspnoea, the predominant symptom in IPF, worsens as disease progresses and leads to exercise limitation and reduced levels of physical activity (PA) [2]. In this respect, objectively measured PA by accelerometry is reduced in IPF patients, compared to healthy individuals, not only in advanced IPF but also in earlier stages [3]. Furthermore, in several cross-sectional studies, PA has been associated to different physiological variables [i.e. forced vital capacity (FVC), carbon monoxide diffusing capacity (DL_CO_) and 6-min walked distance (6MWD)], as well as patient-reported outcomes such as dyspnoea, fatigue and quality of life [3–5]. However, other relevant parameters such as muscle strength or psychological factors have not been explored in detail and, information regarding their contribution or impact on PA in IPF patients is under-researched.

Additionally, there are few longitudinal studies that have examined changes of PA over time in IPF patients [6, 7]. These studies have observed a significant annual decline in PA and have highlighted that this decline could be disproportionate to changes in classical physiological variables, such as lung function and exercise capacity. Moreover, the effects of PA in mortality or progression in IPF are controversial. Bahmer et al. [6] reported an independent association between a lower baseline PA (i.e. steps/day) adjusted by age, sex and antifibrotic therapy, and a higher 3 year mortality risk. By contrast, in a more recent study, neither baseline daily step count (DSC) nor its 12 month decline were associated with long-term survival [7].

The primary aims of our study were: (a) to investigate the relationship between baseline PA and a wide range of variables, including lung function, exercise capacity, dyspnoea, anxiety and depression symptoms, health-related quality of life (HRQoL) and muscle strength in IPF, and (b) to assess longitudinal changes in their PA over 12 months. The secondary aim was to evaluate the impact of PA changes on progression free-survival.

## Methods

### Study population

Patients with IPF diagnosis were consecutively recruited from a specialized ILD clinic in five tertiary-teaching hospitals. Exclusion criteria were relevant comorbidities which prevented PA measurement or the six-minute walking test (6MWT) performance, clinical worsening or hospital admission during two months prior to inclusion, severe pulmonary hypertension detected by echocardiogram [8], treatment with corticosteroid therapy, diagnosis of an active neoplasia or being included in a rehabilitation program.

### Study design

**Fig. 1:**
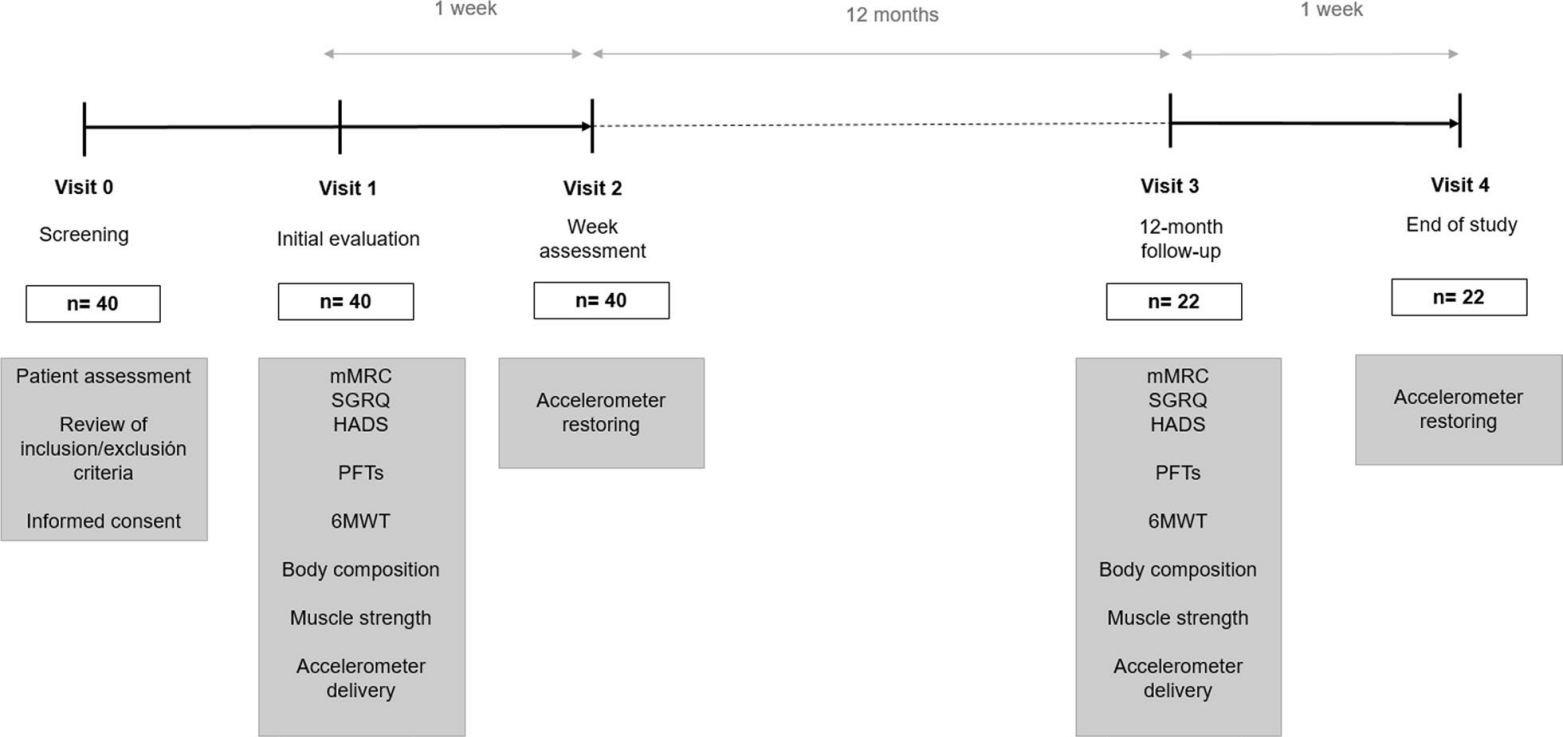
Study flowchart. *mMRC* Modified medical research council; *SGRQ* St. George respiratory questionnaire; *HADS* Hospital anxiety and depression scale; *PFTs* Pulmonary function tests; *6MWT* Six-minute walking test

This is a multicentre prospective study divided into two phases (Fig. 1). Firstly, a cross-sectional evaluation was performed including clinical variables collection such as demographic, (Gender-Age-Physiology) GAP index, body mass index (BMI), Charlson comorbidity index and diagnosis and treatment received. Pulmonary function (PFTs), exercise capacity, respiratory and limb muscle strength and body composition were assessed and dyspnoea, anxiety, depression and HRQoL questionnaires were completed. Moreover, PA was assessed with an accelerometer. A follow-up evaluation was performed at 12 months.

The study was carried out according to the principles of the Declaration of Helsinki for human investigations and approved by the local Ethics Committee with allocation number 2017/7241/I. All participants signed the appropriate informed consent prior to their inclusion.

### Study variables

#### PA measurement

Participants were provided with a triaxial accelerometer (SenseWear Pro2 Armband, SWA; Body Media, Pittsburgh, PA, USA) and were instructed to wear the monitoring device on their left triceps for 23 h a day, for 7 consecutive days except for the time spent in personal hygiene [9]. The minimum recording time for the analysis was defined as at least 3 days, recording more than 70% of the daytime (8–22 h), excluding the first and last day of the record [10].

Parameters of PA included DSC, daily minutes of moderate-to-vigorous PA (MVPA ≥ 3 metabolic equivalents [METS]) and sedentary time (< 1.5 METS) [10, 11]. PA level (PAL: total daily energy divided by sleeping energy expenditure) was also registered and categorized. An inactive person was defined as having a PAL < 1.40, whereas a sedentary person as having a PAL 1.40–1.69, and an active person as having a PAL ≥ 1.70 [12].

#### Dyspnoea, HRQoL and anxiety and depression

The modified Medical Research Council dyspnoea scale (mMRC) [13], the St. George Respiratory Questionnaire (SGRQ) [14] and the Hospital Anxiety and Depression Scale (HADS) [15] were completed as indicated.

#### Lung function and exercise capacity

PFTs including forced spirometry (EasyOne, NDD Medical Technologies, Zurich, Switzerland) static pulmonary volumes (body plethysmography), DL_CO_ (MasterLab, Jaeger, Würzburg, Germany) [16, 17] and the 6MWT were performed according to international guidelines [18]. Arterial blood gas measurement at room air was also performed (RapidLab500, Siemens, Erlangen, Germany).

#### Body mass and composition

Anthropometric variables including BMI and fat-free mass index (FFMI) (acquired by bioelectrical impedance; BODYSTAT 1500, Bodystat Ltd, Isle of Man, UK) were obtained.

#### Respiratory and peripheral muscle strength

Respiratory muscle strength [maximum inspiratory pressure (MIP), maximum expiratory pressure (MEP)] both measured at the mouth, as well as peripheral muscle strength (hand grip: JAMAR 030J1, Chicago, IL, USA) and isometric quadriceps maximum voluntary contraction (QMVC: BIOPAC dynamometer, BIOPAC Systems, Schooner, CA, USA) were assessed following previously described methodology [19]. The highest value of three manoeuvres was recorded. Data were expressed as absolute values and using validated reference equations [20–22].

### Follow-up evaluation

The study participants were assessed after 12 months by repeating the complete protocol. Progression-free survival was assessed up to 24 months after inclusion, and data were obtained from institutional records. A relative decline of FVC > 10% and/or DL_CO_ > 15% during the 24 month follow-up were considered as disease progression [23, 24].

### Statistical analysis

Categorical variables were expressed as number and percentages, whereas continuous variables as mean (standard deviation, SD), or median (25th and 75th percentiles, P_25_-P_75_) when normality assumption was not fulfilled. A multivariate linear regression model was built for each PA variable (DSC, time in MVPA and sedentary time). Potential predictors including BMI, FFMI, FVC (%pred.), DL_CO_ (%pred.), 6MWT (distance and SpO_2_), respiratory and peripheral muscle strength, degree of dyspnoea, SGRQ and HAD score were identified from the literature. Covariates with a p-value < 0.1 were entered in the model, and successively excluded if not associated with the outcome (*p* < 0.05). Finally, the most parsimonious model that still explained the data was built for each outcome. Longitudinal changes in PA were tested by paired *t*-test or Wilcoxon signed-rank test as appropriate. Progression free-survival analysis according to PA changes was analysed by the Kaplan–Meier method. Cox proportional hazard models were performed, adjusted for gender, age and FVC (%pred.), for progression or mortality outcomes. A cut-off point of change of DSC was calculated by maximally selected rank statistic and performed with the web-based tool Cutoff Finder [25]. This method takes jointly into account the marker, the event as well as time to event. A *p*-value < 0.05 was considered as statistically significant. The analysis was performed using the IBM SPSS statistics pack for Windows (Version 23.0, IBM Corp., Armonk, NY, USA).

## Results

### Study participants

Forty participants with IPF were enrolled from July 2017 to December 2019 (Table 1).

**Table 1:** Clinical characteristics of the study population (n = 40)

	Total population (n = 40)
*Demographic*	
Age (years)	71.4 (6.5)
Gender: male	30 (75)
Smoking status: current or former smoker	29 (72.5)
Working status: active	3 (7.5)
GAP index (stage I/II/III), (%)	35/40/25
*Diagnosis and treatment*	
Time since diagnosis (months), median (p25–p75)	18.5 (11.2–42.6)
Ambulatory oxygen therapy	6 (15)
Antifibrotic therapy	30 (75)
Charlson comorbidity index, median (p25–p75)	4 (3–6)
*Lung function*^‡^	
FVC (% pred.)	79.2 (19.4)
TLC (% pred.)	70.6 (14.6)
DL_CO_ (% pred.)	44.7 (14.4)
PaO_2_ (mm Hg)	81.3 (8.7)
*Exercise capacity (6MWT)*	
Distance (m)	451.6 (92.7)
Distance (% pred.)	96.2 (18.6)
Basal SpO_2_ (%)	95.1 (2.4)
Mean SpO_2_ (%)	90.1 (5.8)
Minimum SpO_2_ (%)	87.5 (6.5)
ΔSpO_2_ (%), median (p25–p75)^§^	7 (4–12.8)
*Muscular strength*	
MIP (% pred.)	87.6 (26.3)
MEP (% pred.)	75.3 (23.7)
Non-dominant hand-grip (kg)	30.8 (9.3)
Non-dominant hand grip (% pred.)	113.4 (19.7)
QMVC (kg)	33.6 (9.9)
QMVC (% pred.)	91.9 (25.2)
*Body mass and composition*	
BMI (kg/m^2^)	27.4 (4.6)
FFMI (kg/m^2^)	17.8 (2.1)
*Symptoms, HRQoL and psychological factors*	
Dyspnoea (mMRC 0–4), median (p25–p75)	1 (0.25–2)
SGRQ score (0–100)	
Total	30.3 (20.1)
Activity	48.2 (24.2)
Symptoms	34.4 (22.4)
Impact	27.5 (20.8)
HAD scale (0–21)	
Anxiety	6 (3.6)
Depression	5 (4.4)

Data are presented as mean (SD) and n (%) unless otherwise specified

*GAP* Gender-age-physiology; *FVC* Forced vital capacity; *TLC* Total lung capacity; *DL*_*CO*_ Carbon monoxide diffusion capacity; *PaO*_*2*_ Arterial oxygen partial pressure; *6MWT* Six-minute walking test; *SpO*_*2*_ Peripheral oxygen saturation; *MIP* Maximum inspiratory pressure; *MEP* Maximum expiratory pressure; *QMVC* Quadriceps maximum voluntary contraction; *BMI* Body mass index; *FFMI* Fat-free mass index; *HRQoL* Health-related quality of life; *mMRC* Modified medical research council; *SGRQ* St. George respiratory questionnaire; *HAD* Hospital anxiety and depression

^§^ΔSpO_2_%_,_ percentage of change between baseline and exercise values

^‡^TLC (n = 31), DL_CO_ (n = 33), PaO_2_ (n = 33)

The mean age (SD) at first evaluation was 71.4 (6.5) years, and 75% were males. The mean (SD) FVC % pred. and DL_CO_ % pred. was 79.2 (19.4) % and 44.7 (14.4) %, respectively. Thirty patients (75%) were classified in GAP stages I-II. In the 6MWT, patients walked a mean distance of 451.6 m (92.7%pred.) and showed a mean SpO_2_ of 90%. Respiratory and peripheral muscle strength were roughly preserved in all subjects. Total mean score in the SGRQ was 30.3 and HAD depression and anxiety score were 6 and 5, respectively.

Of the 40 patients studied at baseline, 22 (55%) completed the follow-up. One patient died before study completion, 2 declined and 1 was included in a rehabilitation program during the follow-up period. The 14 remaining subjects were not included, as the 12 month PA measurement was to be completed during the COVID-19 pandemic, and we considered PA and other clinical data could be biased by the lockdown. Accordingly, all PA measurements were performed prior to COVID-19 pandemic to avoid confounders.

### Baseline PA parameters and their predictors

At baseline, subjects performed 5765 (3442) daily steps and spent 64 (44) minutes/day in MVPA. The mean sedentary time was 725 (44) minutes/day. According to PAL, 45.9% of the patients were inactive, 43.2% sedentary and 10.8% active. A lower 6MWD, lower QMVC, and a higher depression score were associated to lower DSC. Sedentary behaviour was associated to the presence of GAP III stage, a BMI ≥ 25 kg/m^2^ and a low QMVC or MIP. A lower time spent in MVPA was related to a lower QMVC or MIP (Table 2).

**Table 2:** Independent predictors of physical activity parameters (daily steps, sedentary time, and time in MVPA) in 40 IPF patients at baseline

Variables	Steps per day		Sedentary time (mins/day)		Time in MVPA (mins/day)	
	β (95% CI)	*p*	β (95% CI)	*p*	(β 95% CI)	*p*
GAP: stage III	–	–	− 27.9 (− 52.6, − 3.2)	0.028	–	–
6MWD (m)	13.7 (4, 23.5)	0.007	–	–	–	–
MIP (%pred.)	–	–	− 0.89 (− 1.3, − 0.47)	< 0.001	0.88 (0.47,1.3)	< 0.001
QMVC (%pred.)	41.7 (7.2, 76.2)	0.019	− 0.57 (− 1.02, − 0.11)	0.016	0.69 (0.23, 1.14)	0.004
BMI: ≥ 25 (kg/m^2^)	–	–	24.5 (0.32, 48.7)	0.047	–	–
HADS score (depression)	− 214.6 (− 416.6, − 12.5)	0.038	–	–	–	–
Adjusted R^2^	0.455		0.561		0.470	

GAP index was dichotomized to GAP I-II and GAP III. BMI was dichotomized by BMI < 25 and ≥ 25

Each column is a single multivariate lineal regression model including as covariates the variables that show a coefficient in each column. Degree of dyspnoea, FVC %pred., Total SGRQ score and HAD anxiety score, were tested as potential predictors, and finally not included because they were not independently related to the outcome, nor modified estimates for the remaining variables

*MVPA* Moderate to vigorous physical activity; *6MWD* Six-minute walked distance; *QMVC* Quadriceps maximum voluntary contraction; *HADS* Hospital anxiety and depression scale; *GAP* Gender-age-physiology; *MIP* Maximum inspiratory pressure; *BMI* Body mass index

### Longitudinal changes in PA

**Fig. 2:**
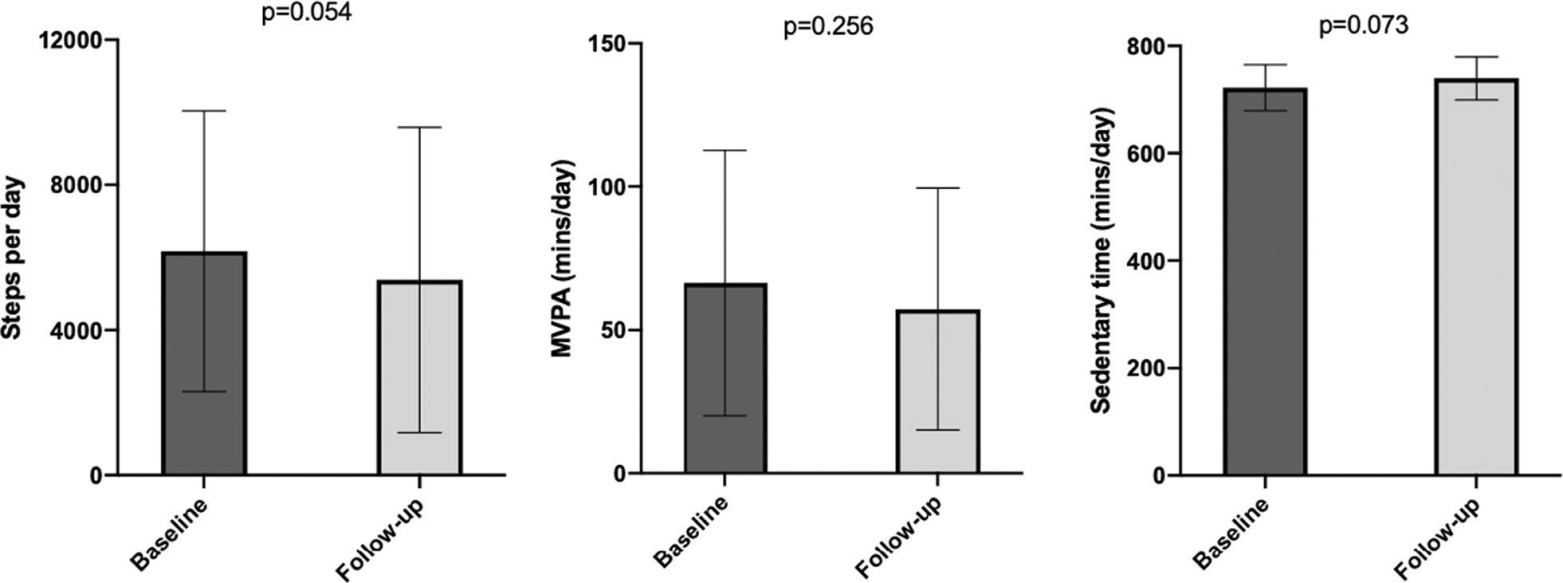
Physical activity parameters at baseline and 12 month follow-up (n = 22). Bars graph show the mean and standard deviation. *PA* Physical activity; *MVPA* Moderate to vigorous physical activity

There were no significant differences in baseline characteristics or PA variables between completers and non-completers except in the anxiety score, which was higher in the non-completer group. DSC decreased by a mean of − 789 (*p* = 0.054). The increase in sedentary time was of 17.5 min/day (*p* = 0.073). No statistically significant changes were observed in MVPA [9.1 min/day; *p* = 0.256], neither in PAL categories (*p* = 0.513) (Fig. 2). Regarding changes in other clinical and functional variables, only DL_CO_ and SpO_2_ during the 6MWT had significant changes over 12 months.

### Progression free-survival analysis

**Fig. 3:**
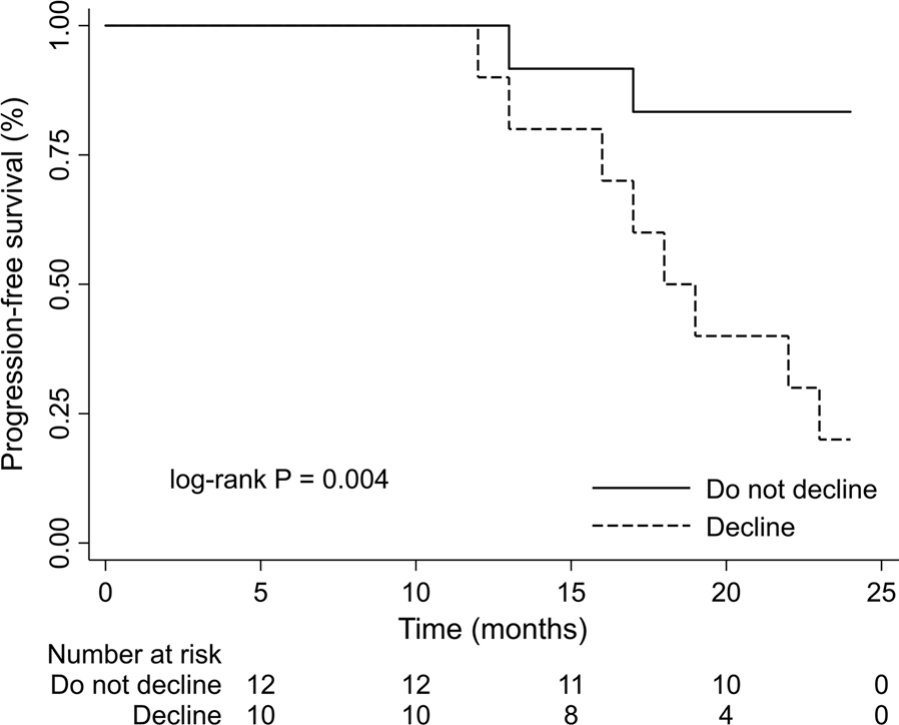
Kaplan–Meier progression-free survival curves at 24 months in IPF patients who decline ≥ 895 steps/day and those who do not decline (n = 22). *IPF* idiopathic pulmonary fibrosis

Patients who died or progressed had a poor lung function, a higher 6MWT desaturation, higher GAP score at baseline, and a higher DSC decline over 12 months than free-progression survivors (1769 vs. − 28; *p* = 0.016). The optimal cut-off point of DSC was -895 steps for predicting the 24 month progression-free survival. Kaplan–Meier curves showed that patients who reduced their PA ≥ 895 steps per day had a lower progression-free survival than those who did not [18.4 months (95%CI, 15.6–21.2) vs. 22.5 (95%CI 20.6–24.4); *p* = 0.004] (Fig. 3). In the Cox regression, a change ≥ to this cut-off point, was associated with a higher progression or mortality risk at 24 months after adjusting for age, sex and FVC (%pred.) [HR 12.1 (95% CI 1.9–78.8); *p* = 0.009].

## Discussion

The main findings of the present study are that quadriceps strength and psychological factors (depression) were identified as novel independent predictors of PA in IPF patients. Moreover, the DSC decline at 12 months was marked; with the 895 daily step threshold being a good discriminant of free-progression survival.

In the present study, PA behaviour was not always consistent with previous literature. In this regard, a higher DSC was related to a higher 6MWD, being consistent with previous studies [3, 5, 26]. By contrast, we observed no association between any PA parameter and lung function, as reported by previous research. A relevant and novel finding of our study was that quadriceps strength was independently associated with the amount and intensity of PA, as well as with sedentary time. The relationship between peripheral muscle dysfunction and reduced PA, is well-documented in other respiratory disorders, but has never been reported specifically in IPF [27, 28]. In ILD, previous research was focused on the role of quadriceps strength on exercise capacity and not on PA, and has the limitation that included heterogeneous groups of ILD patients [29, 30]. Factors such as ageing, hypoxia, oxidative stress, malnutrition and deconditioning due to reduced PA levels, could be associated to potential peripheral muscle dysfunction in IPF, but studies determining its specific causes and pathophysiological mechanisms are lacking [31].

In a similar direction, our results evidenced that a lower MIP was related to a lower time spent in MVPA and a higher sedentary time. It is well established that patients with ILD present a progressive decrease in lung function, lung compliance and an increase in elastic load, secondary to fibrosis. Consequently, the predominant symptom is exertional dyspnoea, which leads to a reduced exercise capacity. Accordingly, several studies have evaluated respiratory muscle strength in ILD, pointing out that the chest mechanical properties remain stable without relationship with lung volume reduction [32, 33] until advanced stages of the disease. Moreover, recent diaphragmatic ultrasound studies, evidence a reduced mobility and diaphragmatic thickness during deep inspiration in ILD subjects compared with healthy individuals, showing no differences in its strength [34]. Our results are in line with the above-mentioned studies, as we found no clear respiratory muscle impairment in our population, although a mild relationship was identified between MIP and PA intensity. One possible explanation could be PA avoidance, leading to deconditioning and muscle wasting. Moreover, mechanical diaphragmatic restraints, could increase the work of breathing resulting in a chronic overload, but this would probably have more impact in muscle endurance that in strength. In this regard, this increased load in respiratory muscles, added to rapid shallow breathing, could increase breathlessness and contribute to reduce PA. In view of the present results, we believe that peripheral and respiratory muscle strength assessment could be of potential interest, and, mainly to direct training strategies to improve exercise tolerance, dyspnoea, and consequently PA.

Additionally, our study showed that a higher depression score was independently associated with a lower DSC. In other chronic lung diseases such as COPD, depression symptoms have been prospectively associated with a reduction in PA at 6 month follow-up [35]. Specifically in ILD, a recent longitudinal study evidenced that depressive symptoms showed a trend towards significance at predicting reduced baseline DSC when adjusting for age, smoking status and lung function [36]. According to our results, psychological status could have an influence on PA in IPF subjects, and therefore, psychological assessment should be considered, to better target areas that can potentially impact on PA levels.

The mean decline in DSC after 12 months in our study was relevant and somewhat different from previous observations. To our knowledge, only two previous studies have focused on changes of PA in IPF, and the population profile, design, and outcomes of each study must be considered when comparing to our data.

Prasad et al. [7] observed a slightly lower decline in DSC after 12 months follow-up in 37 IPF patients. A possible confounder in this study could be the inclusion of lung transplant candidates; and their participation in rehabilitation programs. In addition, Bahmer et al. [6] reported almost a 50% decline in DSC in 3 years, of 23 survivors of an initial cohort of 46 IPF patients. Surprisingly, the annual decline in PA was higher than in our study, despite our patients had similar lung function and a lower baseline PA. Nevertheless, their study provided no information about other PA parameters. Another relevant aspect observed in our study was that the PA decline was not accompanied by significant changes in other physiological parameters, such as FVC or 6MWD. This could be partially explained by the short follow-up period and clinical profile of our population (i.e. mild FVC impairment, preserved 6MWD, and a high percentage of patients treated with antifibrotic therapy). Together, our observations are in line with previous studies that support the idea that the PA decline could be prior to pulmonary function or exercise capacity deterioration, being dependent of other factors as disease progresses, and therefore its longitudinal evaluation could be a complementary tool when evaluating IPF progression [6].

Finally, we evaluated the prognostic value of PA changes over 12 months. To our knowledge, our study is the first to show a threshold for longitudinal changes of DSC (measured by accelerometer) in predicting progression or mortality. There is little and controversial information on the effects of PA in robust outcomes in IPF, and it focuses mainly on baseline assessment of PA. Bahmer et al. [6] reported an independent association between a lower baseline DSC (adjusted by age, sex and antifibrotic therapy) and a higher risk of mortality at 3 years. Moreover, Shingai et al. [37] demonstrated that DSC was a good predictor of 1 year mortality in 87 IPF patients at the time of diagnosis and, additionally they first established a cut-off point (3473 steps) for predicting mortality. By contrast, Prasad et al. [7] observed no association between baseline daily steps count or its decline over 12 months with better long-term survival.

Some limitations of our study should be mentioned. Firstly, the relatively small sample size, especially during the follow-up. However, the percentage of patients lost to follow-up is comparable to previous publications and furthermore there were no differences between those lost and those that completed the follow-up. Further and larger prospective studies to validate the prognostic role of the 895 DSC threshold should be considered. Secondly, the present study cannot establish the direction of the relationships among PA and its predictors. Thirdly, our study population came from a specific geographical area and our results cannot be generalized to countries with different cultural determinants. Despite these limitations, our study had many strengths. It is multicentric and has included a wide range of variables as potential PA predictors, reflecting physiological, psychological and QoL aspects in IPF patients. Finally, we explored other predictors of PA variables beyond DSC, such as PA intensity and sedentary behaviour.

## Conclusions

In summary, our study identifies muscle strength and psychological factors as novel PA predictors in IPF, being both modifiable factors with interventions. These findings suggest potential aspects that can be targeted to improve PA in IPF patients from the diagnosis, and support the need for early inclusion in rehabilitation programs, including respiratory and peripheral muscle training. Moreover, psychological assessment should be considered. Finally, it describes change in DSC as a potential progression or mortality predictor in IPF, suggesting that PA monitoring could be a complementary marker of progression to lung function and exercise capacity, which should be considered in clinical practice. Further longitudinal studies are needed to study the predictors of PA changes and in its prognostic role in IPF, beyond traditional prognostic factors.

## Supplementary Information

**Additional file 1: Table S1.** General characteristics of subjects that completed and not completed follow-up.

**Additional file 2: Table S2.** Longitudinal changes in lung function, exercise capacity, muscle strength, body mass and composition, dyspnoea, quality of life and psychological factors in IPF patients (n=22).

**Additional file 3: Table S3.** General characteristics at baseline of subjects that completed follow-up by progression-free survival** at 24 months (n=22).

**Additional file 4: Table S4.** Longitudinal characteristics (changes in parameters) * by progression-free survival** at 24 months.

## Data Availability

The datasets supporting the conclusions of this article are included within the article (and its additional files). Further enquiries can be directed to the corresponding author.

## Abbreviations

6MWD: Six-minute walked distance
6MWT: Six-minute walking test
BMI: Body mass index
DL_CO_: Carbon monoxide diffusing capacity
DSC: Daily step count
FFMI: Fat-free mass index
FVC: Forced vital capacity
GAP: Gender-age-physiology
HADS: Hospital anxiety and depression scale
HRCT: High resolution computed tomography
ILD: Interstitial lung disease
IPF: Idiopathic pulmonary fibrosis
MEP: Maximum expiratory pressure
METS: Metabolic equivalents
MIP: Maximum inspiratory pressure
mMRC: Modified medical research council
MVPA: Moderate to vigorous physical activity
PA: Physical activity
PAL: Physical activity level
PFTs: Pulmonary function tests
QMVC: Quadriceps maximum voluntary contraction
HRQoL: Health-related quality of life
SGRQ: Saint George respiratory questionnaire
