# Supplementary material for: Predictors and changes of physical activity in idiopathic pulmonary fibrosis
Source: BMC Pulm Med. 2022 Sep 9;22:340. doi: 10.1186/s12890-022-02134-4 (PMC9461180; doi:10.1186/s12890-022-02134-4)
Supplement: Supplementary file 1 — Additional file 1: Table S1. General characteristics of subjects that completed and not completed follow-up. [file 12890_2022_2134_MOESM1_ESM.docx]

**Additional file 1.**

**Table S1. General characteristics of subjects that completed and not completed follow-up**

|  | Follow-up completed  (n=22) | Follow-up not completed  (n= 18) | p value |
| --- | --- | --- | --- |
| **Demographic** |  |  |  |
| Age (years) | 72.7 (6.1) | 69.8 (6.8) | 0.163 |
| Gender: male | 17 (77.3) | 13 (72.2) | 0.731 |
| Smoking habit: Current or former smoker | 16 (72.7) | 13 (72.3) | 0.988 |
| Working status: active | 2 (9.1) | 1 (5.6) | 0.673 |
| Time since diagnosis  (months), median (p25-p75) | 20.9 (15.3-41.7) | 17 (11-50.3) | 0.510 |
| Ambulatory oxygen therapy | 3 (13.6) | 3 (16.7) | 0.789 |
| Antifibrotic therapy | 17 (77.3) | 13 (72.2) | 0.294 |
| Charlson comorbidity index, median (p25-p75) | 3.5 (2.75-6) | 4 (3-6) | 0.492 |
| **Lung function**  FVC (% pred.)  TLC (% pred.) | 76.7 (18.9)  69.8 (13) | 82.3 (20.2)  71.7 (17) | 0.376  0.732 |
| DL_CO_ (% pred.) | 43.9 (13.4) | 45.6 (16) | 0.740 |
| PaO_2_ (mm Hg) | 82.2 (6.2) | 80.1 (11.9) | 0.517 |
| **Exercise capacity, 6MWT**  Distance (m)  Distance (%pred.) | 452.8 (78.8)  98.3 (16.3) | 450.1 (109.7)  93.6 (21.2) | 0.928  0.432 |
| Basal SpO_2_ (%)  Mean SpO_2_ (%) | 95.36 (1.56)  90.5 (5.4) | 94.83 (3.14)  89.4 (6.5) | 0.492  0.581 |
| Minimum SpO_2_ (%),  ΔSpO_2_ (%), median (p25-p75)^*^ | 88.23 (6.03)  7 (4-11.25) | 86.56 (7.11)  6.5 (3.75-20) | 0.426  0.717 |
| **Muscular strength, mean SD**  MIP (%pred.) | 91.3 (27.3) | 83.1 (24.9) | 0.341 |
| MEP (%pred.) | 81.4 (23.3) | 67.9 (22.6) | 0.072 |
| Non-dominant hand-grip (kg)  Non-dominant hand grip (%pred.) | 31 (9.45)  114.9 (20.3) | 30.67 (9.35)  111.7 (19.3) | 0.912  0.618 |
| QMVC (kg)  QMVC (%pred.) | 35.09 (10.4)  97.3 (20.9) | 31.83 (9.2)  85.4 (28.8) | 0.306  0.139 |
| **Body mass and composition**  BMI (kg/m^2^) | 26.9 (3.5) | 27.9 (5.6) | 0.497 |
| FFMI (kg/m^2^) | 17.75 (1.62) | 17.95 (2.68) | 0.770 |
| **Symptoms, HRQoL and psychological factors**  Dyspnoea (mMRC 0-4), median (p25-p75) | 1 (0.75-2) | 1 (0-2) | 0.925 |
| SGRQ score (0-100)  Total  Activity  Impact  Symptoms | 34.8 (16.5)  49.56 (17.46)  27.19 (19.67)  30.39 (15.04) | 35.3 (24.2)  46.52 (31.08)  27.8 (22.8)  39.3 (28.9) | 0.945  0.706  0.931  0.229 |
| Anxiety (HAD, 0-21) | 4.76 (2.7) | 7.5 (3.95) | **0.016** |
| Depression (HAD, 0-21) | 4.05 (4.17) | 6 (4.55) | 0.171 |
|  |  |  |  |
|  |  |  |  |

Data are presented as mean (SD) and n (%) unless otherwise specified.

^*^ ΔSpO_2_ %_,_ percentage of change between baseline and exercise values.

***Abbreviations:*** PA, physical activity; FVC, forced vital capacity; TLC, total lung capacity; DL_CO_, carbon monoxide diffusion capacity; PaO_2_: arterial oxygen partial pressure; 6MWT, six-minute walking test; SpO_2_, peripheral oxygen saturation; MIP, maximum inspiratory pressure; MEP, maximum expiratory pressure; QMVC, quadriceps maximum voluntary contraction; BMI, body mass index; FFMI, fat-free mass index; HRQoL, health-related quality of life; mMRC, modified Medical Research Council; SGRQ, St. George Respiratory Questionnaire; HAD, Hospital Anxiety and Depression.
