# Supplementary material for: Predictors and changes of physical activity in idiopathic pulmonary fibrosis
Source: BMC Pulm Med. 2022 Sep 9;22:340. doi: 10.1186/s12890-022-02134-4 (PMC9461180; doi:10.1186/s12890-022-02134-4)
Supplement: Supplementary file 2 — Additional file 2: Table S2. Longitudinal changes in lung function, exercise capacity, muscle strength, body mass and composition, dyspnoea, quality of life and psychological factors in IPF patients (n=22). [file 12890_2022_2134_MOESM2_ESM.docx]

**Additional file 2.**

**Table S2 . Longitudinal changes in lung function, exercise capacity, muscle strength, body mass and composition, dyspnoea, quality of life and psychological factors in IPF patients (n=22)**

| Variable | At Baseline | At 12 months follow-up | Change (%)^*^ | p value |
| --- | --- | --- | --- | --- |
| Lung function^#^ |  |  |  |  |
| FVC (%pred.) | 75.9 (19.3) | 74.6 (21.6) | -1.7 | 0.332 |
| DL_CO_ (%pred.) | 44.4 (13.6) | 39.8 (16.5) | **-10.3** | **0.005** |
| Exercise capacity (6MWT) |  |  |  |  |
| Distance (m) | 452.8 (78.8) | 438.2 (77.9) | -3.2 | 0.111 |
| Distance (%pred.) | 98.3 (16.3) | 96.1 (15.9) | -2.2 | 0.249 |
| SpO_2_ mean (%) | 91.7 (3.7) | 89.7 (5.1) | **-2.2** | **0.016** |
| Desaturation (%), median (p25-p75) | -7 (4-11.25) | -8 (4.75-14.5) | **2.1** | **0.022** |
| Muscular strength |  |  |  |  |
| MIP (%pred.) | 91.3 (27.3) | 89.3 (16.4) | -2.2 | 0.645 |
| MEP (%pred.) | 81.4 (23.3) | 75.9 (20) | -6.7 | 0.206 |
| Non-dominant hand-grip (%pred.) | 114.5 (20.7) | 109.7 (21.2) | -4.2 | 0.258 |
| QMVC (%pred.) | 97.3 (20.9) | 98.7 (34.9) | 1.4 | 0.817 |
| Body mass and composition |  |  |  |  |
| BMI (kg/m^2^) | 26.9 (3.5) | 26.7 (2.9) | - 0.7 | 0.609 |
| FFMI (kg/m^2^) | 17.8 (1.6) | 16.9 (1.6) | - 5 | 0.063 |
| Dyspnoea, HRQoL and psychological factors |  |  |  |  |
| Dyspnoea (mMRC), median (p25-p75) | 1 (0.75-2) | 1 (1-2) | - 0 | 0.317 |
| SGRQ (score)  *Total*  *Activity*  *Impact*  *Symptoms* | 34.4 (16.8)  49.3 (17.9)  26.6 (20)  30.4 (15.4) | 35.1 (14.8)  53.2 (16)  25.1 (18.2)  30 (20.5) | 2  7.9  -5.6  -1.3 | 0.745  0.205  0.562  0.914 |
| HAD (score)  *Anxiety*  *Depression* | 4.55 (2.89)  3.86 (4.17) | 4.09 (2.77)  4.14 (3.50) | - 10.1  7.2 | 0.285  0.480 |

Data are presented as mean (SD) unless otherwise specified

^*^Relative change: (follow-up parameter- baseline parameter)/baseline parameter

^#^There is missing values for DLco (n=4)

***Abbreviations:*** FVC, forced vital capacity; DLco, carbon monoxide diffusion capacity; 6MWT, 6-minute walking test; SpO_2,_ peripheral oxygen saturation_;_ MIP, maximum inspiratory pressure; MEP, maximum expiratory pressure; QMVC; quadriceps maximum voluntary contraction; BMI, body mass index; FFMI; fat-free mass index; HRQoL, health-related quality of life; HAD, Hospital Anxiety and Depression scale; SGRQ, Saint George Respiratory Questionnaire; mMRC, modified medical research council.
