# Supplementary material for: Predictors and changes of physical activity in idiopathic pulmonary fibrosis
Source: BMC Pulm Med. 2022 Sep 9;22:340. doi: 10.1186/s12890-022-02134-4 (PMC9461180; doi:10.1186/s12890-022-02134-4)
Supplement: Supplementary file 3 — Additional file 3: Table S3. General characteristics at baseline of subjects that completed follow-up by progression-free survival** at 24 months (n=22). [file 12890_2022_2134_MOESM3_ESM.docx]

**Additional file 3.**

**Table S3. General characteristics at baseline of subjects that completed follow-up by progression-free survival** at 24 months (n=22).**

| Variable | Progression- free survivors  (n=12) | Non-survivor or progression  (n=10) | p value |
| --- | --- | --- | --- |
| Demographic |  |  |  |
| Age (years) | 73.8 (7.2) | 71.4 (4.6) | 0.385 |
| GAP index (score) | 1.67 (0.65) | 2.5 (0.53) | **0.004** |
| Lung function |  |  |  |
| FVC (% pred.) | 85.3 (19.7) | 66.4 (11.8) | **0.015** |
| DL_CO_ (% pred.) | 51.3 (12) | 34.6 (8.6) | **0.040** |
| Exercise capacity (6MWT) |  |  |  |
| Distance (m) | 456 (81.7) | 449 (79.4) | 0.841 |
| Distance (%pred.) | 101.3 (15.7) | 94.8 (17.1) | 0.368 |
| Mean SpO_2_ (%) | 93.2 (2.9) | 87.6 (6) | **0.013** |
| Minimum SpO_2_ (%) | 91.4 (3.4) | 84.4 (6.4) | **0.040** |
| ΔSpO_2_ (%)^*^ | 4.7 (3.1) | 12.5 (8.1) | **0.005** |
| Muscular strength |  |  |  |
| MIP (%pred.) | 93.7 (26.2) | 88.2 (30) | 0.663 |
| MEP (%pred.) | 85.1 (24.9) | 77 (21.7) | 0.431 |
| Non-dominant hand grip (%pred.) | 112.4 (18.6) | 117.8 (22.8) | 0.551 |
| QMVC (%pred.) | 98.6 (24.8) | 95.6 (16.2) | 0.743 |
| Body mass and composition |  |  |  |
| BMI (kg/m^2^) | 25.9 (2.5) | 28.1 (4.3) | 0.145 |
| FFMI (kg/m^2^) | 17.3 (1.6) | 18.3 (1.6) | 0.156 |
| Symptoms, HRQoL and psychological factors |  |  |  |
| Dyspnoea (mMRC) | 1 (0.85) | 1.6 (1.1) | 0.160 |
| SGRQ (score)  *Total*  *Activity*  *Symptoms*  *Impact* | 34.3 (18.6)  44.6 (19.5)  33.4 (16)  28.3 (21.8) | 35.3 (14.8)  55 (13.8)  27 (14)  25.9 (18.1) | 0.890  0.178  0.344  0.787 |
| HADS (score)  *Anxiety*  *Depression* | 4.2 (2.8)  3.2 (3.9) | 5.4 (2.8)  5 (4.4) | 0.327  0.332 |
| Physical activity |  |  |  |
| Steps per day | 6909 (3996) | 5282 (3714) | 0.338 |
| MVPA (mins/day) | 75.2 (43.2) | 55.7 (50) | 0.339 |
| Sedentary time (mins/day) | 718 (40) | 730 (45) | 0.544 |
| PAL | 1.5 (0.16) | 1.38 (0.2) | 0.218 |

Data are presented as mean (SD).

^*^ ΔSpO_2_ %_,_ percentage of change between baseline and exercise values.

***Abbreviations:*** FVC, forced vital capacity; DL_CO_, carbon monoxide diffusion capacity; 6MWT, 6-minute walking test; SpO_2_, peripheral oxygen saturation; MIP, maximum inspiratory pressure; MEP, maximum expiratory pressure; QMVC; quadriceps maximum voluntary contraction; BMI, body mass index; FFMI; fat-free mass index; HRQoL, health-related quality of life; mMRC, modified Medical Research Council; SGRQ, Saint George Respiratory Questionnaire; HAD, Hospital Anxiety and Depression scale; MVPA, moderate-vigorous physical activity; PAL, physical activity level

**defined as survival with no relative decline of FVC > 10% and/or DL_CO_ > 15%
