# Supplementary material for: Predictors and changes of physical activity in idiopathic pulmonary fibrosis
Source: BMC Pulm Med. 2022 Sep 9;22:340. doi: 10.1186/s12890-022-02134-4 (PMC9461180; doi:10.1186/s12890-022-02134-4)
Supplement: Supplementary file 4 — Additional file 4: Table S4. Longitudinal characteristics (changes in parameters) * by progression-free survival** at 24 months. [file 12890_2022_2134_MOESM4_ESM.docx]

**Additional file 4.**

**Table S4.**  **Longitudinal characteristics (changes in parameters) * by progression-free survival** at 24 months**

| Variable | Progression- free survivors  (n=12) | Non-survivor or progression  (n=10) | p value |
| --- | --- | --- | --- |
| Exercise capacity (6MWT) |  |  |  |
| Distance (m) | - 1.83 (18) | - 30 (55.6) | 0.112 |
| Distance (% pred.) | 0.75 (4.8) | - 5.9 (11.5) | 0.083 |
| Basal SpO_2_ (%) | 0 (0.74) | 0.6 (2.7) | 0.464 |
| Mean SpO_2_ (%) | - 1.45 (1.9) | - 2.3 (4.5) | 0.564 |
| Minimum SpO_2_ (%) | -1.2 (2.6) | -2.5 (4.3) | 0.397 |
| Muscular strength |  |  |  |
| MIP (%pred.) | -1.4 (22.8) | -2.9 (17.1) | 0.873 |
| MEP (%pred.) | - 6.7 (27.3) | 2.8 (28.2) | 0.429 |
| Non-dominant hand grip (%pred.) | - 2.7 (19.8) | - 7.6 (18.6) | 0.572 |
| QMVC (% pred.) | -8.3 (17.2) | -13 (35.5) | 0.080 |
| Body mass and composition |  |  |  |
| BMI (kg/m^2^) | 2 (1.3) | -0.6 (1.5) | 0.207 |
| FFMI (kg/m^2^) | -1.15 (1.6) | -0.39 (2.24) | 0.368 |
| Symptoms, HRQoL and psychological factors |  |  |  |
| Dyspnoea (mMRC) | 2.7 (0.46) | 0 (8.1) | 0.353 |
| SGRQ (score)  *Total*  *Activity*  *Symptoms*  *Impact* | - 1.5 (10.3)  4.1 (14.1)  0.2 (19.6)  -5.7 (11.1) | 3.3 (6)  3.7 (12.9)  -1.2 (14.5)  3.7 (9.3) | 0.236  0.947  0.867  **0.059** |
| HADS (score)  *Anxiety*  *Depression* | -0.6 (1.5)  0.18 (0.98) | -0.3 (2.5)  0.4 (2.5) | 0.709  0.792 |
| Physical activity |  |  |  |
| Steps per day | 28.3 (1649) | -1769 (1534) | **0.016** |
| MVPA (mins/day) | -4.6 (43.4) | -14.5 (27.2) | 0.539 |
| Sedentary time (mins/day) | 22.1 (30.1) | 12.4 (54) | 0.611 |
| PAL | 0.07 (0.1) | - 0.09 (0.12) | **0.088** |

Data are presented as mean (SD)

*Change in parameters = follow-up parameter – baseline parameter

**defined as survival with no relative decline of FVC > 10% and/or DL_CO_ > 15%

***Abbreviations:*** 6MWT, 6-minute walking test; SpO_2_**_,_** peripheral oxygen saturation; MIP, maximum inspiratory pressure; MEP, maximum expiratory pressure; QMVC; quadriceps maximum voluntary contraction; BMI, body mass index; FFMI; fat-free mass index; HRQoL, health-related quality of life; mMRC, modified Medical Research Council; SGRQ, Saint George Respiratory Questionnaire; HAD, Hospital Anxiety and Depression scale; MVPA, moderate-vigorous physical activity; PAL, physical activity level.
